# Supplementary material for: Evaluation of Six Years of Appropriateness Level of Blood Transfusion in a Pediatric Ward
Source: Int J Environ Res Public Health. 2023 Jan 17;20(3):1700. doi: 10.3390/ijerph20031700 (PMC9914791; doi:10.3390/ijerph20031700)
Supplement: Supplementary file 1 [file ijerph-20-01700-s001.zip › ijerph-2126746-supplementary.pdf]

Table S1. NHS clinical indication for Blood Component Transfusion

Component	Disease	Clinical indication
Red Blood Cells		
	Haemoglobinopathies: Sick Cells Syndrome (SCS) or thalassaemia	Acute Chest Syndrome in SCS<br>Stroke or Priapism inSCS<br>Maintaining an average Hb of 12 g/dl<br>Maintaining a pretransfusion Hb of 9–10g/dl
	Haemopoietic Stem Cell Transplant (HSCT) and oncologic disease	Symptomatic patients with Hb values <7 g/dl
	Surgery Procedures	Surgery Procedures in SCS<br>Cardiac surgery
Platelet transfusion		
	Thrombocytopenia as a result of reduced production	Platelet count <10×10⁹/l<br>Platelet count <20×10⁹/l and one or more of the following:<br>Severe mucositis<br>Disseminated intravascular coagulation (DIC)<br>Anticoagulant therapy<br>Platelets likely to fall <10×10⁹/l before next evaluation<br>Risk of bleeding due to a local tumour infiltration<br>Platelet count 20–40×10⁹/l and one or more of the following:<br>DIC in association with induction therapy for leukaemia<br>Extreme hyperleucocytosis<br>Prior to lumbar puncture or central venous line insertion
Fresh Frozen Plasma		
	Active bleeding	Disseminated intravascular coagulation (DIC) with bleeding<br>Bleeding following cardiac surgery<br>Major haemorrhage

Table S2. Demographic features and agreement with NHS criteria of Blood transfusion performed (N=147)

N° ID	Gender	Age	NHS Criteria	Agreement with NHS criteria
1	M	5-10	Infection in Sick Cell Syndrome	Clinical decision mediated
2	M	15-30	Other	Not Agreed
3	M	5-10	Anemia in autoimmune syndrome	Not Agreed
4	M	0-5	Infection in Sick Cell Syndrome	Not Agreed
5	M	0-5	Infection in Sick Cell Syndrome	Not Agreed
6	M	0-5	Infection in Sick Cell Syndrome	Not Agreed
7	M	0-5	Anemia	Not Agreed
8	F	15-30	Infection in Sick Cell Syndrome	Clinical decision mediated
9	F	15-30	Anemia in Sick Cell Syndrome	Clinical decision mediated
10	M	0-5	Autoimmune disease	Not Agreed
11	M	15-30	Anemia	Not Agreed
12	M	5-10	Anemia in genetic syndrome	Not Agreed
13	F	0-5	Neoplasms	Not Agreed
14	F	0-5	Sick Cell Syndrome (SCS) with Acute chest Syndrome	Not Agreed
15	M	0-5	Anemia in infectious diseases	Clinical decision mediated
16	F	10-15	Anemia in active bleeding	Clinical decision mediated

17	F	10-15	Anemia in active bleeding	Clinical decision mediated
18	M	0-5	Hemolitic Uremic Syndrome	Not Agreed
19	F	0-5	Hemolitic Uremic Syndrome	Clinical decision mediated
20	F	0-5	Hemolitic Uremic Syndrome	Clinical decision mediated
21	M	10-15	Active bleeding in autoimmune syndrome	Clinical decision mediated
22	M	10-15	Active bleeding in autoimmune syndrome	Clinical decision mediated
23	M	15-30	Neoplasms	Not Agreed
24	M	0-5	Neoplasms	Not Agreed
25	M	0-5	Autoimmune anemia	Clinical decision mediated
26	F	0-5	Autoimmune anemia	Clinical decision mediated
27	F	10-15	Hemolitic Uremic Syndrome	Clinical decision mediated
28	F	10-15	Hemolitic Uremic Syndrome	Clinical decision mediated
29	F	10-15	Hemolitic Uremic Syndrome	Clinical decision mediated
30	F	15-30	Anemia in thrombosis	Not Agreed
31	F	15-30	Anemia in thrombosis	Not Agreed
32	M	5-10	Hemolitic Uremic Syndrome	Clinical decision mediated
33	M	5-10	Hemolitic Uremic Syndrome	Clinical decision mediated
34	M	0-5	Anemia in Sick Cell Syndrome	Not Agreed
35	M	0-5	Anemia in Sick Cell Syndrome	Not Agreed
36	F	15-30	Hematologic malignancy	Agreed
37	F	15-30	Hematologic malignancy	Agreed
38	M	0-5	Anemia in genetic syndrome	Clinical decision mediated
39	F	0-5	Anemia in active bleeding	Clinical decision mediated
40	M	0-5	Anemia in Sick Cell Syndrome	Not Agreed
41	F	15-30	Anemia in active bleeding	Clinical decision mediated
42	F	15-30	Active bleeding in autoimmune disease	Clinical decision mediated
43	F	0-5	AKI in genetic syndrome	Clinical decision mediated
44	F	0-5	Anemia in Sick Cell Syndrome	Not Agreed
45	F	0-5	Hemolitic Uremic Syndrome	Clinical decision mediated
46	F	0-5	Hemolitic Uremic Syndrome	Clinical decision mediated
47	M	5-10	Neoplasms	Clinical decision mediated
48	M	5-10	Neoplasms	Not Agreed
49	F	10-15	Liver transplantation	Clinical decision mediated
50	F	10-15	Liver transplantation	Clinical decision mediated
51	F	10-15	Liver transplantation	Clinical decision mediated
52	F	10-15	Coagulation genetic disease	Clinical decision mediated
53	F	10-15	Coagulation genetic disease	Clinical decision mediated
54	F	10-15	Coagulation genetic disease	Clinical decision mediated
55	F	10-15	Coagulation genetic disease	Clinical decision mediated
56	F	10-15	Coagulation genetic disease	Clinical decision mediated
57	F	10-15	Coagulation genetic disease	Clinical decision mediated
58	F	10-15	Coagulation genetic disease	Clinical decision mediated
59	F	10-15	Coagulation genetic disease	Clinical decision mediated
60	F	10-15	Coagulation genetic disease	Clinical decision mediated
61	F	10-15	Coagulation genetic disease	Clinical decision mediated
62	F	10-15	Coagulation genetic disease	Clinical decision mediated
63	F	10-15	Coagulation genetic disease	Clinical decision mediated
64	F	10-15	Coagulation genetic disease	Clinical decision mediated
65	F	10-15	Coagulation genetic disease	Clinical decision mediated
66	F	10-15	Coagulation genetic disease	Clinical decision mediated
67	F	10-15	Coagulation genetic disease	Clinical decision mediated
68	F	10-15	Coagulation genetic disease	Clinical decision mediated
69	F	10-15	Coagulation genetic disease	Clinical decision mediated
70	F	10-15	Coagulation genetic disease	Clinical decision mediated
71	F	10-15	Coagulation genetic disease	Clinical decision mediated
72	F	10-15	Coagulation genetic disease	Clinical decision mediated
73	F	10-15	Coagulation genetic disease	Clinical decision mediated
74	F	15-30	Coagulation genetic disease	Clinical decision mediated

75	F	15-30	Coagulation genetic disease	Clinical decision mediated
76	F	15-30	Coagulation genetic disease	Clinical decision mediated
77	F	15-30	Coagulation genetic disease	Clinical decision mediated
78	F	15-30	Coagulation genetic disease	Clinical decision mediated
79	M	0-5	Hemolitic Uremic Syndrome	Not Agreed
80	F	0-5	Hemolitic Uremic Syndrome	Clinical decision mediated
81	F	0-5	Hemolitic Uremic Syndrome	Clinical decision mediated
82	M	15-30	Anemia in Sick Cell Syndrome	Agreed
83	M	15-30	Anemia in Sick Cell Syndrome	Agreed
84	M	0-5	Anemia in Malaria	Not Agreed
85	M	10-15	Anemia in Genetic Syndrome	Not Agreed
86	M	0-5	Hemolytic anemia	Clinical decision mediated
87	F	15-30	Autoimmune disease	Not Agreed
88	F	15-30	Autoimmune disease	Not Agreed
89	F	15-30	Other	Clinical decision mediated
90	F	15-30	Other	Clinical decision mediated
91	M	0-5	Hemolitic Uremic Syndrome	Clinical decision mediated
92	M	0-5	Hemolitic Uremic Syndrome	Clinical decision mediated
93	F	0-5	Hemolytic anemia	Clinical decision mediated
94	F	0-5	Anemia in autoimmune disease	Clinical decision mediated
95	F	0-5	Anemia in autoimmune disease	Clinical decision mediated
96	F	5-10	Anemia in autoimmune disease	Clinical decision mediated
97	F	5-10	Autoimmune disease	Not Agreed
98	F	5-10	Anemia in genetic syndrome	Clinical decision mediated
99	F	5-10	Anemia in genetic syndrome	Clinical decision mediated
100	F	5-10	Anemia in genetic syndrome	Clinical decision mediated
101	F	15-30	Autoimmune disease	Not Agreed
102	F	15-30	Autoimmune disease	Not Agreed
103	F	15-30	Anemia in autoimmune disease	Clinical decision mediated
104	F	15-30	Anemia in autoimmune disease	Clinical decision mediated
105	F	15-30	Anemia in autoimmune disease	Clinical decision mediated
106	F	15-30	Anemia in autoimmune disease	Clinical decision mediated
107	F	15-30	Anemia in autoimmune disease	Clinical decision mediated
108	F	15-30	Anemia in autoimmune disease	Clinical decision mediated
109	F	15-30	Anemia in autoimmune disease	Clinical decision mediated
110	M	5-10	Sick Cell Syndrome	Not Agreed
111	M	5-10	Acute chest pain in Sick Cell Syndrome	Agreed
112	M	5-10	Acute chest pain in Sick Cell Syndrome	Agreed
113	M	5-10	Infection in Sick Cell Syndrome	Clinical decision mediated
114	M	5-10	Infection in Sick Cell Syndrome	Clinical decision mediated
115	M	5-10	Other	Not Agreed
116	F	0-5	Other	Not Agreed
117	F	10-15	Anemia in genetic syndrome	Clinical decision mediated
118	F	0-5	Other	Not Agreed
119	M	15-30	Anemia in Sick Cell Syndrome	Not Agreed
120	M	0-5	Other	Not Agreed
121	M	0-5	Active bleeding in genetic syndrome	Clinical decision mediated
122	M	0-5	Neoplasms	Not Agreed
123	M	5-10	Neoplasms	Clinical decision mediated
124	F	0-5	Neoplasms	Clinical decision mediated
125	F	0-5	Neoplasms	Clinical decision mediated
126	F	5-10	Anemia and fever in autoimmune disease	Clinical decision mediated
127	F	10-15	Anemia in emolitic disease	Clinical decision mediated
128	F	10-15	Anemia in emolitic disease	Clinical decision mediated
129	M	10-15	Hemolitic Uremic Syndrome	Clinical decision mediated
130	M	10-15	Hemolitic Uremic Syndrome	Clinical decision mediated
131	M	0-5	Autoimmune disease	Not Agreed
132	F	15-30	Active bleeding in genetic disease	Clinical decision mediated

133	M	0-5	Anemia in Sick Cell Syndrome	Clinical decision mediated
134	F	15-30	Autoimmune disease	Not Agreed
135	F	15-30	Autoimmune disease	Not Agreed
136	F	15-30	Autoimmune disease	Not Agreed
137	F	15-30	Autoimmune disease	Not Agreed
138	M	15-30	Active bleeding in autoimmune disease	Clinical decision mediated
139	M	15-30	Active bleeding in autoimmune disease	Clinical decision mediated
140	M	15-30	Active bleeding in autoimmune disease	Clinical decision mediated
141	M	15-30	Active bleeding in autoimmune disease	Clinical decision mediated
142	M	15-30	Neoplasms	Not Agreed
143	M	15-30	Neoplasms	Not Agreed
144	F	0-5	Other	Not Agreed
145	F	0-5	Anemia in autoimmune disease	Clinical decision mediated
146	F	10-15	Other	Not Agreed
147	F	0-5	Hemolitic Uremic Syndrome	Clinical decision mediated
